# Metabolic pathways are affected differently in *Saccharomyces cerevisiae* thiol peroxidase mutants during redox stress with hydrogen peroxide

**DOI:** 10.1186/gb-2011-12-s1-p16

**Published:** 2011-09-19

**Authors:** Amy L Olex, Brian M Westwood, Leslie B Poole, Jacquelyn S Fetrow

**Affiliations:** 1 Department of Computer Science, Wake Forest University, Winston-Salem, NC 27109, USA; 2 Department of Molecular Genetics and Genomics, Wake Forest University School of Medicine, Winston-Salem, NC 27157, USA; 3 Department of Biochemistry, Wake Forest University School of Medicine, Winston-Salem, NC, 27157, USA; 4 Department of Physics, Wake Forest University, Winston-Salem, NC 27109, USA

## Background

Thiol peroxidases have been conserved throughout evolution and are found in almost every known organism from bacteria to humans. These proteins play a key role in maintaining redox homeostasis and have been implicated in other processes such as cell signaling and sensing hydrogen peroxide and passing this signal along to transcription factors. To gain a better understanding of the role that each thiol peroxidase plays in redox regulation on a global level, Fomenko and colleagues [1] performed a series of microarray experiments in which different combinations of the genes encoding the eight thiol peroxidases (three glutathione peroxidase homologs (Gpx) and five peroxiredoxins (Prx)) present in yeast were knocked out, including one mutant (8-Δ) in which all eight peroxidases were removed. Surprisingly, all of the mutants, including 8-Δ, were viable and could withstand redox stresses; however, they were unable to activate or repress transcriptional events in response to hydrogen peroxide treatment, which was most evident in the 8-Δ mutant. In our work, network analysis was used to gain a better understanding of the biological networks whose gene expression is affected by these mutations.

## Methods

Microarray data (provided by [1]) was processed for input into the Cytoscape plug-in jActiveModules. Active sub-networks for select mutants were identified using all yeast interactions found in the Kyoto Encyclopedia of Genes and Genomes (KEGG) [2] as the background network (including protein-protein, metabolic and gene expression interactions). Nodes in each sub-network were input into the Database for Annotation, Visualization and Integrated Discovery (DAVID) [3] to identify which KEGG pathways were present.

## Results

Two hundred and six genes appeared in one or more of the active sub-networks. Only seven genes were present in the sub-networks of all strains. These were a known oxidative stress-induced aldose reductase (*GRE3*), four putative aryl-alcohol dehydrogenases (*AAD3*, *AAD6*, *AAD10* and *AAD14*), a mitochondrial aldehyde dehydrogenase (*ALD4*) and a xylulokinase (*XKS1*). All of the genes were upregulated on average by 6- to 12-fold in all strains, except for 8-Δ with a 1.5-fold average upregulation and 5Prx-Δ with a 3-fold average upregulation.

Many metabolic pathways were affected by the knockouts; the pathway types affected depended on which peroxidase gene was knocked out. This result suggests that different thiol peroxidases may have a significant and specific impact on the regulation of metabolic pathways during oxidative stress.

Surprisingly, the Gpx3-Δ active sub-network was similar to the Gpx1-Δ and Gpx2-Δ sub-networks. Gpx3 is known to sense hydrogen peroxide and pass that signal along to transcription factors; thus, it was expected that this subnetwork would differ from that of the other Gpx mutants. Additionally, our results showed that amino acid metabolism, biosynthesis and degradation pathways were active in wild-type cells but were present in few mutant strains.

## Conclusions

The results of this work indicate that thiol peroxidases, along with playing a key role in maintaining redox homeostasis, may also play a significant role in the regulation of metabolic pathways in yeast, thus illuminating the global role that thiol peroxidases play in oxidative stress.
